# Disturbed B-lymphocytes selection in autoimmune lymphoproliferative syndrome

**DOI:** 10.1186/2194-7791-2-S1-A23

**Published:** 2015-07-01

**Authors:** Aleš Janda, Klaus Schwarz, Mirjam van der Burg, Werner Vach, Hanna Ijspeert, Myriam Ricarda Lorenz, Magdeldin Elgizouli, Kathrin Pieper, Paul Fisch, Joachim Hagel, Raquel Lorenzetti, Maximilian Seidl, Joachim Roesler, Fabian Hauck, Elisabetta Traggiai, Carsten Speckmann, Anne Rensing-Ehl, Stephan Ehl, Hermann Eibel, Marta Rizzi

**Affiliations:** Centre for Chronic Immunodeficiency, (CCI), University Medical Centre, Freiburg, Germany; Centre for Pediatrics and Adolescent Medicine, University Medical Centre, University of Freiburg, Germany; Institute for Transfusion Medicine, University of Ulm, Ulm, Germany; Institute for Clinical Transfusion Medicine and Immunogenetics Ulm, German Red Cross Blood Service Baden-Württemberg - Hessen, Ulm, Germany; Department of Immunology, Erasmus MC, University Medical Center Rotterdam, Rotterdam, The Netherlands; Institute of Medical Biometry and Medical Statistics, University Medical Center, Freiburg, Germany; Department of Pathology, Molecular Pathology, University Medical Center, University of Freiburg, Germany; Department of Pediatrics, University Clinic Carl Gustav Carus, Dresden, Germany; Department of Pediatric Hematology, Oncology and Immunology, Dr von Hauner Children's Hospital, Ludwig-Maximilians-University, Munich, Germany; Novartis Institute for Biomedical Research, Basel, Switzerland; Clinic for Rheumatology and Clinical Immunology, University Medical Centre, University of Freiburg, Germany

## Meeting abstract

Autoimmune lymphoproliferative syndrome (ALPS) is characterized by lymphoproliferative disease, autoimmune cytopenias and increased susceptibility to lymphoid malignancies. Central to the pathophysiology of the disease are defects in the FAS signaling leading to impaired lymphocyte homeostasis. Most of the patients harbor heterozygous germline or somatic mutations in *FAS*. The hallmark of the disease is the impaired FAS-mediated apoptosis of activated T cells and presence of atypical "double-negative" T cells (CD3+TCRalpha/beta+CD4-CD8-). While FAS is essential for deletion of autoreactive B cells in the germinal center in murine models, the role of FAS in human B cell selection and development of autoimmunity in patients carrying *FAS* mutation is unclear.

We analyzed patients with somatic *FAS* mutation or germline *FAS* mutation plus somatic loss-of-heterozygosity allowing to compare the fate of B cells with impaired versus normal FAS signaling within the same individual. We found in the class-switched memory B cells: 1) accumulation of *FAS-* mutated B cells, 2) a failure to enrich single V genes and in single V-D, D-J gene combinations of the B cell receptor variable region, 3) increased frequency of variable regions with higher content of positively charged amino acids and longer CDR3 and 4) maintenance of polyreactive specificities. Importantly, *FAS*-deficient switched memory B cells showed increased rates of somatic hypermutation. Our data uncover a defect in B cell selection in ALPS patients and indicate a role for B cell dysregulation in the pathogenesis of autoimmunity and B-cell lymphoma in ALPS patients.

